# ID2-ETS2 axis regulates the transcriptional acquisition of pro-tumoral microglia phenotype in glioma

**DOI:** 10.1038/s41419-024-06903-3

**Published:** 2024-07-18

**Authors:** Guillermo Vázquez-Cabrera, Martin Škandík, Noémie Roncier, Farah Real Oualit, Mireia Cruz De Los Santos, Austeja Baleviciute, Mathilde Cheray, Bertrand Joseph

**Affiliations:** 1 Institute of Environmental Medicine, Karolinska Institutet, Stockholm, Sweden; 2 Department of Oncology Pathology, Karolinska Institutet, Stockholm, Sweden; 3 Center for Neuromusculoskeletal Restorative Medicine, Shui On Centre, Wan Chai, Hong Kong

**Keywords:** CNS cancer, Microglial cells

## Abstract

Glioblastoma is a highly aggressive brain tumour that creates an immunosuppressive microenvironment. Microglia, the brain’s resident immune cells, play a crucial role in this environment. Glioblastoma cells can reprogramme microglia to create a supportive niche that promotes tumour growth. However, the mechanisms controlling the acquisition of a transcriptome associated with a tumour-supportive microglial reactive state are not fully understood. In this study, we investigated changes in the transcriptional profile of BV2 microglia exposed to C6 glioma cells. RNA-sequencing analysis revealed a significant upregulation of microglial inhibitor of DNA binding 1 (*Id1*) and *Id2*, helix-loop-helix negative transcription regulatory factors. The concomitant regulation of microglial ETS proto-oncogene 2, transcription factor (ETS2)-target genes, *i.e*., *Dusp6*, *Fli1*, *Jun*, *Hmox1*, and *Stab1*, led us to hypothesize that ETS2 could be regulated by ID proteins. In fact, ID2-ETS2 protein interactions increased in microglia exposed to glioma cells. In addition, perturbation of the ID2-ETS2 transcriptional axis influenced the acquisition of a microglial tumour-supportive phenotype. *ID2* and *ETS2* genes were found to be expressed by the tumour-associated microglia isolated from human glioblastoma tumour biopsies. Furthermore, *ID2* and *ETS2* gene expressions exhibited inverse prognostic values in patients with glioma in cohorts from The Cancer Genome Atlas. Collectively, our findings indicate that the regulation of ETS2 by ID2 plays a role in the transcriptional regulation of microglia in response to stimuli originating from glioblastoma cells, information that could lead to developing therapeutic strategies to manipulate microglial tumour-trophic functions.

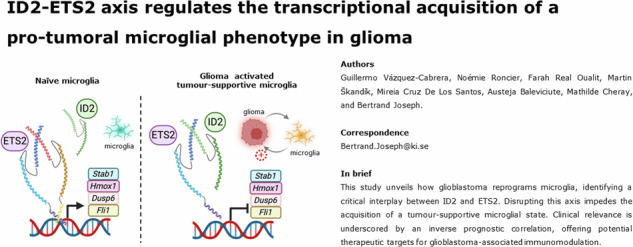

## Introduction

High-grade malignant gliomas (HGG), such as glioblastoma (GB) remain one of the challenges of today’s oncology due to their significant mortality and morbidity. The intrinsic capacity of these HGG to infiltrate the surrounding brain tissue, which impedes complete neurosurgical resection, contributes significantly to the failure of current therapeutic treatments, and predictably results in high rates of early recurrence. Despite multimodal therapy with the concomitant use of radiation and adjuvant treatment (*e.g*. temozolomide, bevacizumab), and the recommended use of alternating electric field therapy to newly diagnosed patients, the overall median survival for patients with GB is approximately 15 months [1]. An emerging view is that if new therapeutic strategies are to be found for this neoplasm, one will have to take into account that GB tumours are heterogeneous with respect to the composition of bona fide tumours cells and with respect to a range of intermingling non-neoplastic cells, including microglia, which also play a vital roles in controlling the course of pathology [2–4].

Microglia, the resident myeloid immune cells of the central nervous system, are reported in mice to derive from primitive erythromyeloid precursors that originate from the yolk sac and populate the brain during early embryonic development [5, 6]. Microglia, upon cues in their microenvironment, acquire different reactive states defined notably by distinct gene expression profiles and with unique functions that contribute to brain development, homoeostasis, as well as pathologies. In fact, the number of unique transcriptome signature used to define different microglial reactive states associated with diseases have been accumulating in the last decade [4, 7–13]. In the context of GB, microglia are recruited in large numbers to the tumour site, to form together with peripherally recruited bone-marrow-derived macrophages, the tumour-associated myeloid cells component of the tumour microenvironment [14]. Glioma cancer cells are able to hijack the microglial transcriptome thereby reprograming these immune cells to exert tumour-trophic functions, hence promoting neoplasm progression [2, 4, 13]. Several factors released from microglia, including growth factors, cytokines and extracellular matrix proteases, have been reported to promote tumour invasiveness and growth [2]. As further evidence of their implication in glioma biology, depletion of microglia in organotypic brain slices inhibits glioma invasiveness [15, 16]. Exacerbating their tumour-supporting phenotype in vivo, for instance *via* the silencing of microglial *CASP3* expression, has been shown to enhance GB tumor growth [14]. In contrast, interventions aiming at the depletion of these tumour-associated myeloid cells through the inhibition of colony-stimulating factor-1 receptor (CSF-1R) whose downstream signalling is required for their survival, have been reported to reduce, at least transiently, GB expansion [17, 18]. However, these studies also highlight that targeting the entire brain myeloid cell population may not be the most successful strategy to combat brain neoplasms such as GB. Since, distinct gene expression profiles have been associated to each of the microglial reactive states, including the tumour-supportive one, the identification of modulators of selective microglial transcriptomic signature, which have the potential to regulate unique microglial function, has recently gained interest.

Therefore, we have decided to further investigate the identification of potential transcriptional regulators that could contribute to the acquisition of a tumour-supportive phenotype by microglia upon their stimulation by glioma cells. Here, we report the identification of inhibitor of DNA binding 2 (ID2, also known as inhibitor of differentiation 2), a helix-loop-helix (HLH) protein and transcriptional regulator, as well as its interacting transcriptional partner, ETS proto-oncogene 2, transcription factor (ETS2) as involved in the acquisition of the microglial reactive state associated with tumour-trophic functions. *ID2* and *ETS2* genes were found to be expressed by the tumour-associated microglia isolated from human GB tumour biopsies. Furthermore, *ID2* and *ETS2* gene expressions exhibited inverse prognostic values for patients with glioma.

## Materials and methods

### Cell culture

BV2 (referred as BV2) mouse microglia cell line (RRID:CVCL_0182; gift of G. Brown, University of Cambridge) [14, 19], and C6 rat glioma cell line (RRID:CVCL_0194; purchased from ATCC, CCL-107™) were cultivated in DMEM + glutamax medium (Gibco) supplemented with 10% FBS (foetal bovine serum Gibco, 10270-106, LOT 42F1490K) and 1% Penicillin/Streptomycin (Gibco). All cell lines were grown in an incubator at 37 °C and 5% CO_2_ and regularly tested for mycoplasma using the Lookout mycoplasma PCR detection kit (Sigma). For segregated coculture experiments, cells were first seeded in 5% FBS medium, BV2 cells on coverslips in 12-well plates and C6 cells in 94 mm diameter Petri dishes or 6 well plates. Thereafter, 24 h after seeding, the coculture experiments were initiated by placing the coverslips with microglial cells into 40 µm cell strainers in the petri dishes containing the glioblastoma cells [14, 20]. The microglia cells were then harvested at specified time points.

### RNA isolation, cDNA synthesis, and qPCR

Total RNA was extracted using the RNeasy Plus Mini Kit (Qiagen). RNA concentrations were quantified using NanoDrop® spectrophotometer (Thermo Fisher Scientific). cDNA was synthesized from 1 µg RNA using Oligo dT, dNTPs, and Superscript IV Reverse Tanscriptase (Invitrogen). qPCR was run on an StepOne plus (Applied Biosystems) using the SYBR™ Green master mix (life technologies), CFX Duet Real-Time PCR System (Biorad) using SsoAdvanced Universal SYBR^®^ Green Supermix (Biorad) and primers listed in Supplementary Table 1. *Actb* (actin beta) gene expression in each sample was used for normalization. Relative Quantification (RQ) results were calculated using ΔΔ*C*_*t*_ method and represented as a fold over control cells.

### LEGENDPlex^TM^ flow cytometry-based multiplex immunoassays

Supernatants from 24 h cocultures performed as described above were collected, centrifuged at (4500 G, 3 min, 4 °C), stored at −80 °C and thereafter stained with the cytometric bead-based assay “LEGENDplex™ Mouse Macrophage/Microglia Panel” (BioLegend, REF: 740846) following the manufacturer protocol. Data acquisition was made by analyzer Sony ID7000. Analysis was performed by “Data Analysis Software Suite for LEGENDplex™” Powered by QOGNIT.

### Library preparation and RNA-sequencing

Total RNA was subjected to quality control with Agilent Tapestation according to the manufacturer’s instructions. Two hundred nanograms of total RNA was subjected to Illumina sequencing and libraries were prepared with the Illumina TruSeq Stranded mRNA kit which includes cDNA synthesis, ligation of adaptors and amplification of indexed libraries. The yield and quality of the amplified libraries was analyzed using Qubit by Thermo Fisher and the Agilent Tapestation. The indexed cDNA libraries were normalized and combined, and the pools were sequenced on the Illumina Hiseq 2000 generating 50 bp single-end reads.

### RNA-Seq data and computational analysis

Basecalling and demultiplexing were performed using Illumina bcl2fastq v2.20.0.422 software with default settings, generating FASTQ files for further downstream mapping and analysis. Differentially expressed genes with significant (<0.01) FDR were used for all further analysis.

### Volcano plot

For the “positive versus negative” comparison, volcano plots were created in GraphPad Prism, which displayed significance and fold change for the dataset together with gene symbols for the most highly regulated genes.

### TRRUST analysis

Transcriptional regulatory relationships unravelled by sentence-based text mining (TRRUST), a bioinformatics tool provided through the Enrichr website (maayanlab.cloud/Enrichr/, [21]) was used to show transcription factors associated with our gene lists of interest, using a cut-off of <0.05 for the *p* value.

### Immunoblotting

Total protein extracts were made directly in Laemmli buffer by scraping of the cells. Samples were sonicated (Diagenode, Bioruptor Pico) and boiled, and proteins were then separated by 10% SDS–polyacrylamide gel electrophoresis and blotted onto 0.2 μm pore-size nitrocellulose membranes (Bio Rad) using the Mini Trans-Blot wet transfer system (Bio Rad). Membranes were blocked with 0.1% Tween 20 (Sigma-Aldrich) and 5% milk in PBS and incubated overnight at 4 °C with indicated primary antibodies (listed in Supplementary Table 2). Membranes were incubated with RDye® secondary antibodies (LI-COR Biosciences) according to the manufacturer’s instructions. Protein bands were visualized using the Odyssey CLx infra-red imaging system (LI-COR Biosciences) equipped with the software Image Studio Lite, version 5.2 (LI-COR Biosciences). All targeted proteins of interest were normalized to the selected housekeeping protein ACTB (actin beta), and intensity of the bands was quantified using ImageJ software. Full immunoblots used for this study are presented in Supplementary Data File 1.

### Gene silencing by transfection of small interfering RNAs pools

Non-targeting control, *Id2* and *Ets2* ON-TARGET plus SMARTpools siRNAs, whose sequences can be found in Supplementary Table 3, were obtained from Dharmacon. Transfection of BV2 cells was carried out with Lipofectamine 3000 (Invitrogen) following the manufacturer’s instructions.

### Transwell migration assay

Eight micrometers-pore width transparent PET membrane inserts (Transwell, Corning) were used to measure cell migration capability. C6 glioma cells were seeded on top of the insert and BV2 microglia were seeded in the lower compartment. Once the experiment was finalized (after 6 or 24 h), the membranes from the inserts were fixed with 4% paraformaldehyde in PBS for 15 min, washed with PBS and carefully cut out with a blade. Later on, the membranes were mounted with ProLong Gold antifade reagent with DAPI (Life technologies) and the nuclei of the migrated cells were counted under fluorescent microscopy using a Zeiss AXIO Observer Inverted Fluorescence Microscope. Analysis of the images was performed using ImageJ.

### In situ proximity ligation assay

BV2 microglial cells were grown on coverslips for 24 h and thereafter cocultured with C6 glioblastoma cells in a segregated coculture system for 2 as previously described hours. BV2 cells were fixed with 4% paraformaldehyde in PBS for 15 min. Cells were permeabilized 10 min in PBS-0.01% Triton X100 and blocked 1 h in PLA blocking buffer. Then, the In situ PLA (Duolink II Detection Reagents Red Kit, Olink Bioscience) was performed according to the manufacturer’s instructions. The samples were mounted using ProLong Gold antifade mounting medium containing DAPI. Fluorescence signal amplification was observed using Zeiss LSM800-airy confocal laser scanning microscopy. Analysis of the images was performed using ImageJ. Antibodies used are listed in Supplementary Table 2.

### Human single-cell RNA-sequencing data from glioblastoma tumours

The *ID2* and *ETS2* gene expression levels in the myeloid, microglial or macrophage cell populations, in the context of human glioblastoma tumours were analyzed taking advantage a publicly available 201,986 single cells RNA-sequencing dataset (Gene Expression Omnibus (GEO) accession number: GSE182109) generated from 18 glioma patients, including 1 astrocytoma, 1 oligodendroglioma, 11 newly diagnosed GB, and 5 recurrent GB [22], whose visualization are accessible via the Singel Cell Portal at the following URL:

https://singlecell.broadinstitute.org/single_cell/study/SCP1985/single-cell-analysis-of-human-glioma-and-immune-cells-identifies-s100a4-as-an-immunotherapy-target-gse182109.

Successive filters were applied on the single cell RNA-sequencing dataset to look exclusively at the newly diagnosed GB (94,450 cells), then at myeloid cells within those tumours (36,998 cells), that were thereafter subdivided into microglia (28,634 cells) and macrophages (8,364 cells).

The *ID2* and *ETS2* gene expression levels in the microglia/macrophage cell populations, in the context of human glioblastoma tumours were analyzed taking advantage a publicly available 7930 single cells RNA-sequencing dataset (Gene Expression Omnibus (GEO) accession number: GSE131928) generated from 28 adult and paediatric GB tumours [23], whose visualization are accessible via the Singel Cell Portal at the following URL:

https://singlecell.broadinstitute.org/single_cell/study/SCP393/single-cell-rna-seq-of-adult-and-pediatric-glioblastoma#study-visualize.

### Survival analysis

Gene expression data (RNA-seq) and clinical data from Cancer Genome Atlas (TCGA) glioblastoma (TCGA-GBM; 617 cases; https://portal.gdc.cancer.gov/projects/TCGA-GBM) and lower-grade Glioma (TCGA-LGG; 516 cases; https://portal.gdc.cancer.gov/projects/TCGA-LGG) cohorts were acquired using UCSCXenaTools, only 694 patients with both survival and transcriptomic data were finally included into R Software (version 4.2.3) [24]. Patient survival was analyzed by Kaplan–Meier method using R package survival. High and low gene expression groups were defined by median value and visualized employing survminer. Differences between the two groups were assessed for significance through log-rank test.

### Statistical analysis

All values are a mean of at least 3 independent biological replicates ± SEM. Statistical analysis was performed using GraphPad Prism (GraphPad Software, Version 10.0), the threshold for statistical significance was considered when the *p* value was less than 0.05.

## Results

### Increased inhibitor of DNA binding protein family members expression is observed in microglia exposed to glioma cells

**Fig. 1:**
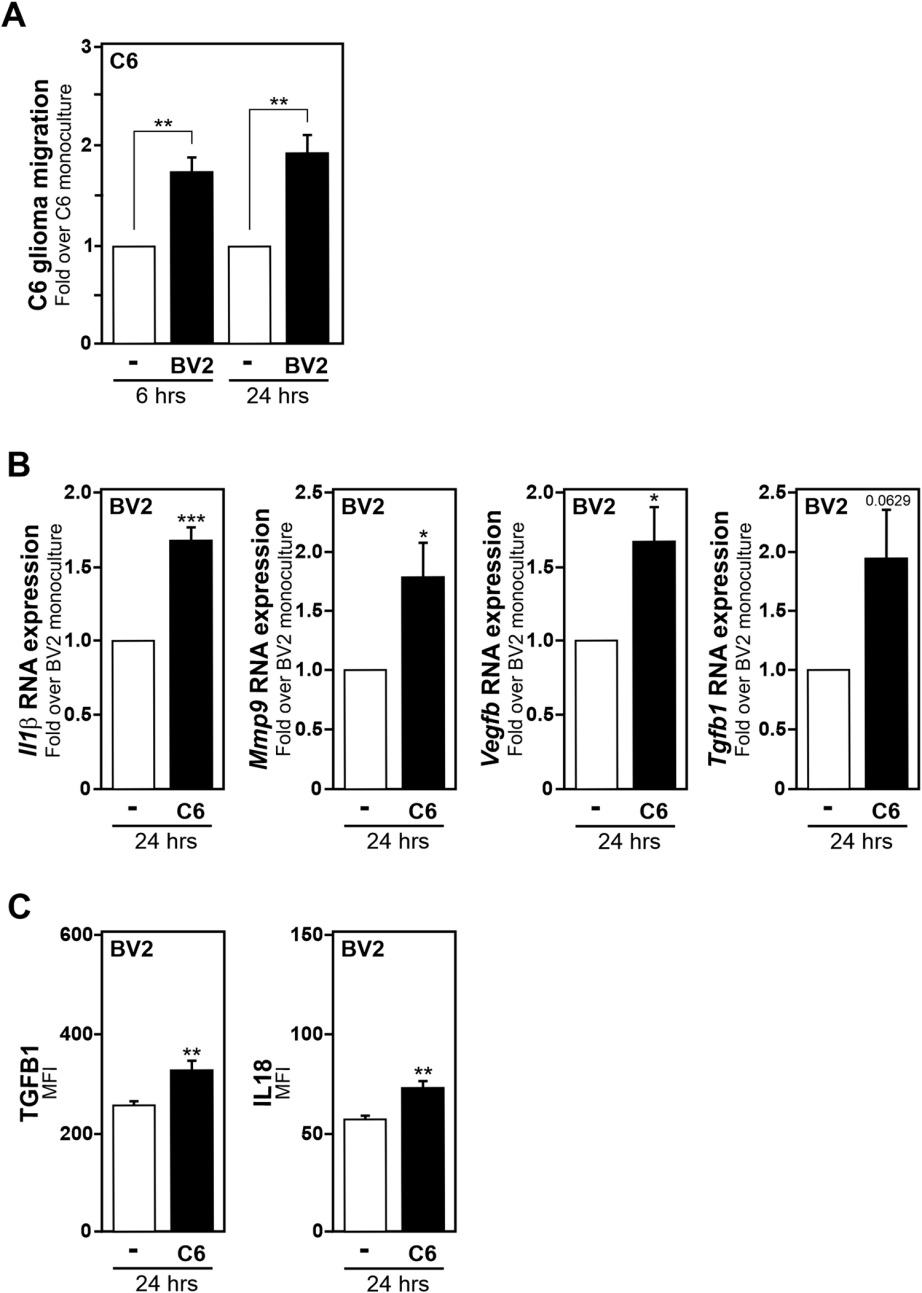
BV2 microglia exposed to C6 glioma cells acquire a tumour-supportive phenotype. **A** Quantification of the migration capacity of C6 glioma cells in transwells with BV2 microglia in the lower compartment. Results are presented relative to the migration of C6 glioma cells alone (-), set as 1. **B** Comparison of *Il1β*, *Mmp9*, *Vegfb* and *Tgfb1* mRNA expression measured by RT-qPCR in BV2 microglia exposed to C6 glioma cells for 24 h as compared to BV2 microglia monoculture, set as 1. **C** Secreted IL18 and TGFB1 (active form) protein levels in 24 h BV2 microglia/C6 glioma cells, as compared to BV2 microglia monoculture, assessed using a flow cytometric bead-based assay. Data, mean fluorescence intensity (MFI) values, are presented as mean ± SEM. Statistics, paired comparisons, were performed with a Student’s *t*-test from at least 3 independent experiments (**A**, *n* = 3; **B**, **C** *n* = 4;). *P* value: *<0.05, **< 0.01, ***< 0.001, n.s. not significant for the indicated comparison.

**Fig. 2:**
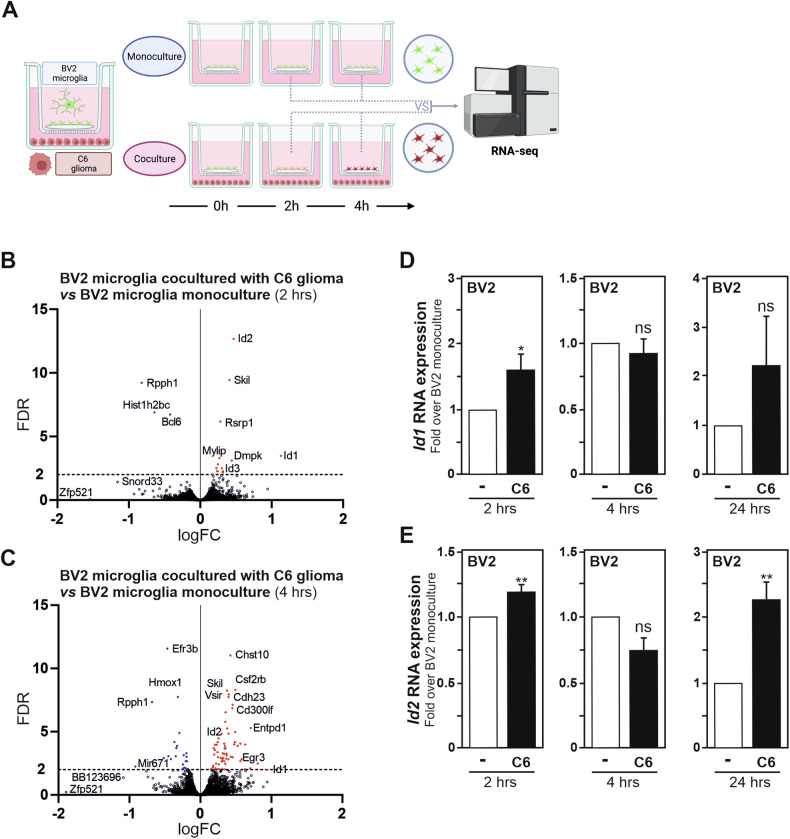
Increased ID protein family members expression is observed in microglia exposed to glioma cells. **A** Illustration of the workflow used for bulk RNA sequencing of BV2 microglia monoculture, compared to BV2 microglia exposed to C6 glioma cells in segregated coculture. Volcano plot of up- and downregulated (FDR < 0.01) genes in BV2 microglia exposed to C6 glioma cells for 2 h (**B**) and 4 h (**C**) as compared to BV2 microglia monoculture. Comparison of *Id1* (**D**) and *Id2* (**E**) mRNA expression measured by RT-qPCR in BV2 microglia exposed to C6 glioma cells for 2, 4 and 24 h as compared to BV2 microglia monoculture, set as 1. Data are presented as mean ± SEM. Statistics, paired comparisons, were performed with a Student’s t-test from at least 3 independent experiments (**B**, **C** *n* = 3; **D**, **E** (2 and 4 h) *n* = 6, **D**, **E** (24 h) *n* = 4). *P* value: *<0.05, **< 0.01, n.s. not significant for the indicated comparison.

To gain further insight into how glioma tumour cells promote the transcriptomic reprogramming of microglia toward a tumour-supportive reactive state, we took advantage of an established rodent in vitro segregated coculture system, in which mouse BV2 microglial cells are exposed to soluble factors originating from rat C6 glioma cells [14, 20]. Upon exposure to C6 glioma cells originating stimuli, BV2 microglia acquire a tumour-supportive phenotype, as demonstrated by their ability to enhance the migration capability of the cancer cells evaluated using a transwell migration assay (Fig. 1A), as well as the expression of genes (*Il1β*, *Mmp9*, *Vegfb* and *Tgfb1*) and secretion of proteins (IL18, TGFB1) associated to a microglial tumour-supportive phenotype (Fig. 1B, C). The genome-wide transcriptomic profiles of BV2 microglia exposed in a segregated coculture setup to C6 glioma cells for 2 and 4 h were investigated by bulk RNA sequencing. The transcriptomes of BV2 monoculture at the identical time points were used as controls for comparison (workflow in Fig. 2A). The generated RNA-seq data revealed BV2 microglia stimulated by C6 glioma cells possess a unique and distinct transcriptomic profile compared to naive BV2 microglia. At the 2-h timepoint, we identified 14 upregulated genes and 3 downregulated genes, within values of FDR < 0.01, in BV2 microglia coculture with C6 glioma cells compared to BV2 microglia (Fig. 2B). At the 4-h timepoint, using the same criteria, 62 upregulated genes and 22 downregulated genes were observed (Fig. 2C).

Among those genes, a significant upregulation in the expression of genes encoding for inhibitors of DNA binding (ID) family proteins, *i.e., Id1*, *Id2* (2 and 4-hour time points) and *Id3* (2-h timepoint), was observed (Fig. 2B, C). ID proteins are a class of HLH transcription regulatory factors that act as dominant-negative antagonists for basic HLH (bHLH) transcription factors, as well as other non-bHLH transcription factors, through the formation of non-functional heterodimers [25, 26]. Hence, ID proteins represent possible essential transcriptional regulators that impact on the acquisition of a microglial tumour-supportive reactive state, and therefore their contribution was further investigated. To confirm the upregulation of *Id* gene expression in C6 glioma cells stimulated-BV2 microglia, the microglial expression of *Id1* and *Id2* mRNAs was examined by quantitative PCR with reverse transcription (RT-qPCR). A transient upregulation for both *Id1* and *Id2* messengers was validated in BV2 microglia, 2 h post segregated coculture with C6 glioma cells, when compared to BV2 microglia monoculture (Fig. 2D, E).

### TRRUST analysis of the glioma cells-induced microglial transcriptome reveals ETS proto-oncogene 2, transcription factor as a potential microglial tumour-supportive phenotype regulator

**Fig. 3:**
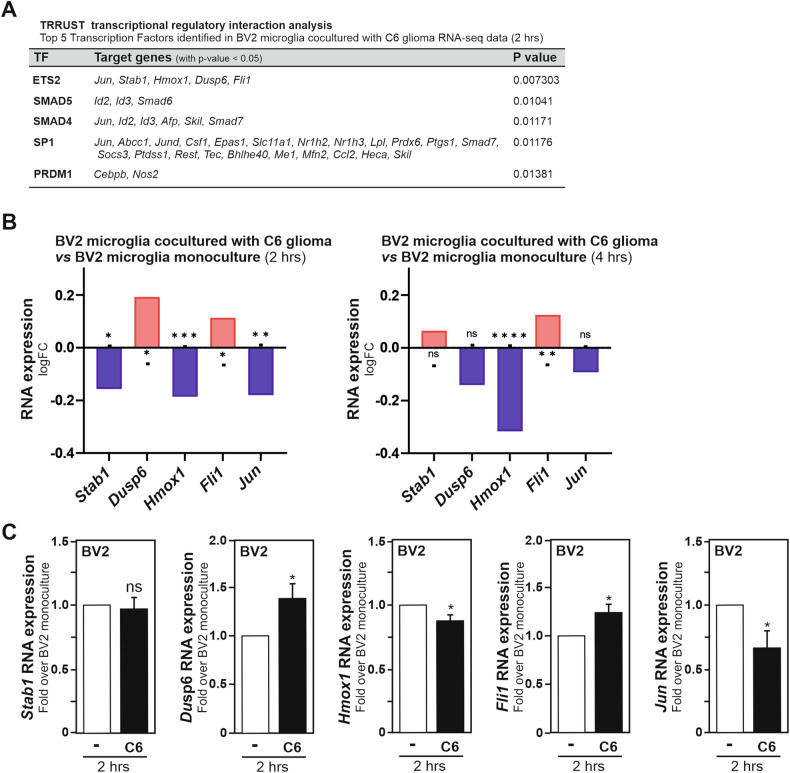
Transcriptome TRRUST analysis of glioma-stimulated microglia reveals ETS2 as a regulator of microglial pro-tumoral phenotype. **A** TRRUST (Transcriptional Regulatory Relationships Unraveled by Sentence-based Text mining) analysis, which allows the identification of transcription factor potentially involved in cell response based on the expression of their target genes, revealed ETS2 as a transcription factor candidate regulated in BV2 microglia after 2 h of segregated coculture with C6 glioma cells. Input genes used exhibited differential expression in BV2 cells cocultured with C6 cells as compared to BV2 cells in monoculture (*p* value < 0,05), significance of association is shown with the respective *p* value. **B** ETS2-target genes mRNA expression obtained from the RNA-seq analysis of BV2 microglia cocultured with C6 glioma cells for 2 and 4 h, as compared to BV2 microglia monoculture. P value obtained from RNAseq analysis. **C** ETS2-target genes mRNA expression measured by RT-qPCR in BV2 microglia exposed to C6 glioma cells for 2 h as compared to BV2 microglia monoculture, set as 1. Data are presented as mean ± SEM. **C** Statistics were performed with a Student’s *t*-test from 4 independent experiments (*Jun*) and 7 independent experiments (*Stab1*, *Dusp6*, *Hmox1*, *Fli1*). P value: *<0.05, **<0.01, ***<0.001, ****<0.0001, n.s. not significant for the indicated comparison.

After having demonstrated in BV2 microglia stimulated by C6 glioma cells, an upregulation of two transcription regulatory cofactors, *i.e*., ID1 and ID2, we thought to identify the transcription factors that could contribute to the acquisition of the microglial tumour-supportive gene expression signature, and whose transcriptional activities could be regulated by interactions with ID proteins. For this purpose, we took advantage of the transcription factor-target interaction database TRRUST (Transcriptional Regulatory Relationships Unraveled by Sentence-based Text mining). TRRUST software allows for the identification of transcription factors potentially involved in cellular responses based on the expression of their target genes; with its current version contains 6552 regulatory relationships of 828 mouse transcription factors with their target genes [21]. This bioinformatics tool was accessed through the Enrichr website [27]. In the RNA-sequencing datasets, 954 genes were found to be significantly differentially expressed, in BV2 microglia cocultured for 2 h with C6 glioma cells when compared to BV2 monoculture, using for the analysis a cut-off of <0.05 for the *p* value. ETS proto-oncogene 2, transcription factor (ETS2) was found to exhibit the most robust statistical significance (*p* value = 0.007303) with the implication of several ETS2-target genes, namely *Jun* (Jun proto-oncogene, AP-1 transcription factor subunit), *Hmox1* (heme oxygenase 1), *Stab1* (stabilin 1), *Dusp6* (dual specificity phosphatase 6) and *Fli1* (Fli1 proto-oncogene, ETS transcription factor) (Fig. 3A). From the RNA-sequencing datasets, the comparative RNA expression for these ETS2-target genes in BV2 microglia cocultured with C6 glioma cells for 2 or 4 h as compared to their respective BV2 monocultures is depicted in Fig. 3B. Significant upregulation of *Dusp6* and *Fli1* gene expressions and downregulation of *Hmox1* and *Jun* gene expressions in BV2 microglia, 2 h post segregated coculture with C6 glioma cells, were confirmed by RT-qPCR analysis (Fig. 3C). Thus, these analyses advocate for the involvement of the transcription factor ETS2 in the regulation of the acquisition of a microglial tumour-supportive phenotype.

### Increased ID2-ETS2 protein–protein interactions are observed in microglia exposed to glioma cells

**Fig. 4:**
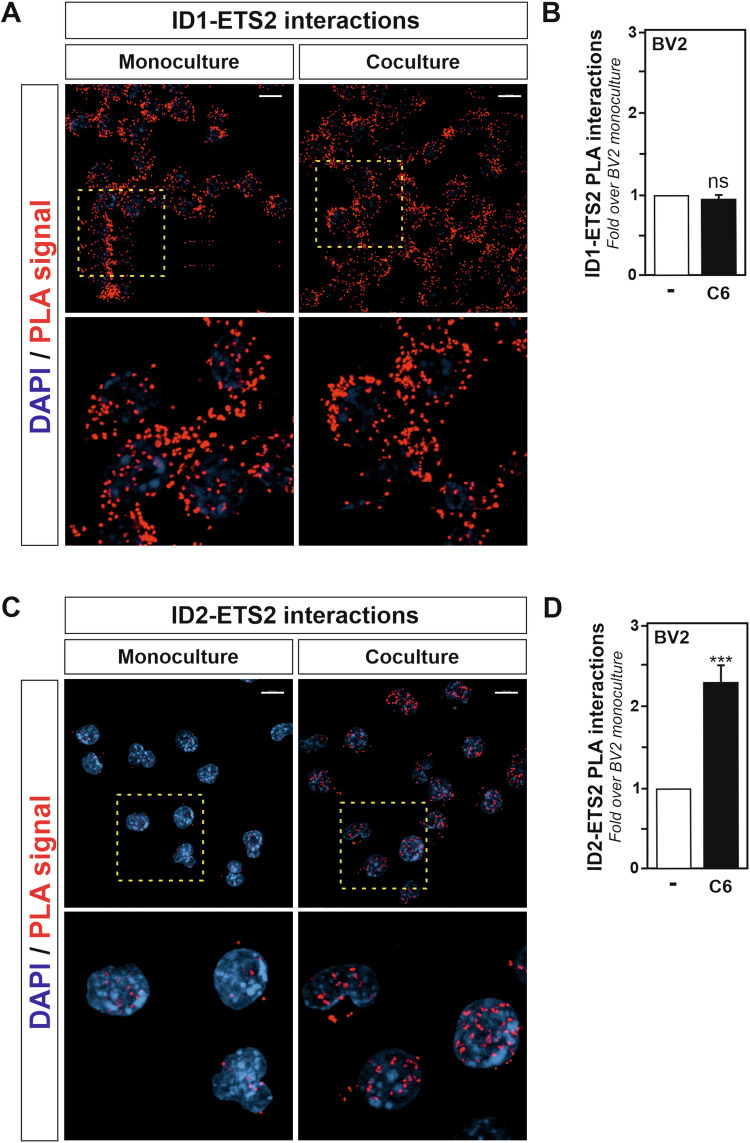
Increased ID2-ETS2 protein-protein interactions are observed in microglia exposed to glioma cells. **A**, **C** Confocal imaging analysis of in situ proximity ligation assay reveals increased protein-protein interaction between ETS2 and ID2 (**C**) but not ID1 (**A**) in BV2 microglia cocultured for 2 h with C6 glioma cells as compared to BV2 alone used as control. DAPI was used for nuclear counterstaining. The bottom picture represents a crop of the above corresponding picture indicated by a yellow square. **B**, **D** Quantification of the PLA experiments displayed in panels (**A**) and (**C**) respectively. Dots were counted and normalized to the number of cells in each picture. The normalized values dots/cell from the BV2 microglia exposed to C6 glioma cells was compared to monocultured BV2 cells, set as 1. Data are presented as mean ± SEM. Statistics were performed with a Student’s t-test from 3 independent experiments. *P* value: ***<0.001. n.s. not significant for the indicated comparison. Scale bars for (**A**, **C**): 10 µm.

ID proteins have been reported to interact with and regulate the transcriptional activities of non-bHLH transcription factors, including members of the ETS transcription factor family of proteins that include ETS2 (reviewed in [25]). For example, ETS and ID proteins are reported to form complexes that regulate transcription, *e.g*., *FOS* (Fos proto-oncogene, AP-1 transcription factor subunit, also known as *c-fos*), *TERT* (telomerase reverse transcriptase) and *CDKN2a* (cyclin-dependent kinase inhibitor 2A, also known as *p16INK4A*) genes [28–30]. To gain further insight into the possible involvement of an ID proteins/ETS2 transcriptional axis in the regulation of microglia activation toward a reactive state supporting tumour growth, protein-protein interactions between ETS2 and ID1 and/or ID2 were investigated. by taking advantage of the in situ proximity ligation assay (PLA) approach. In situ PLA, performed to detect ID1-ETS2 protein-protein interactions (Fig. 4A, B) or ID2-ETS2 protein-protein interactions (Fig. 4C, D), revealed that both types of interactions exist in the unstimulated BV2 microglia. However, only the ID2-ETS2 protein-protein interactions were found to significantly increased in the BV2 microglia after 2 h exposure to C6 glioma cells originating stimuli. Therefore, whereas both *Id1* and *Id2* genes expression appear to be upregulated in microglial cells exposed to glioma cells, and ID1 and ID2 proteins can form protein-protein complexes with ETS2 in microglia, only a transcriptional axis formed by ID2 and ETS2 seems to be of relevance for the acquisition of the microglial tumour-supportive phenotype.

### Perturbation of the ID2-ETS2 axis affects the acquisition of a microglial tumour-supportive phenotype

To gain further understanding of the importance of the ID2-ETS2 transcriptional regulatory axis in the acquisition of the C6 glioma cells induced tumour-supportive phenotype in BV2 microglia, *Id2* or *Ets2* gene expression were transiently repressed in BV2 microglia prior to their exposure to C6 glioma cells. Transfection of BV2 microglia with a pool of small-interfering RNAs targeting *Id2* gene expression led to a significant downregulation of *Id2* mRNA expression, but did not affect *Id1* gene expression, as assessed by RT-qPCR analysis (Supplementary Fig. 1A and 1B). Likewise, RT-qPCR analysis reveals that BV2 microglia transfected with a pool of small-interfering RNAs targeting *Ets2* gene expression exhibited a significant downregulation of *Ets2* mRNA expression level (Supplementary Fig. 1C). In addition, immunoblotting analysis confirmed the downregulation of ETS2 at the protein level (Supplementary Fig. 1D, E).

**Fig. 5:**
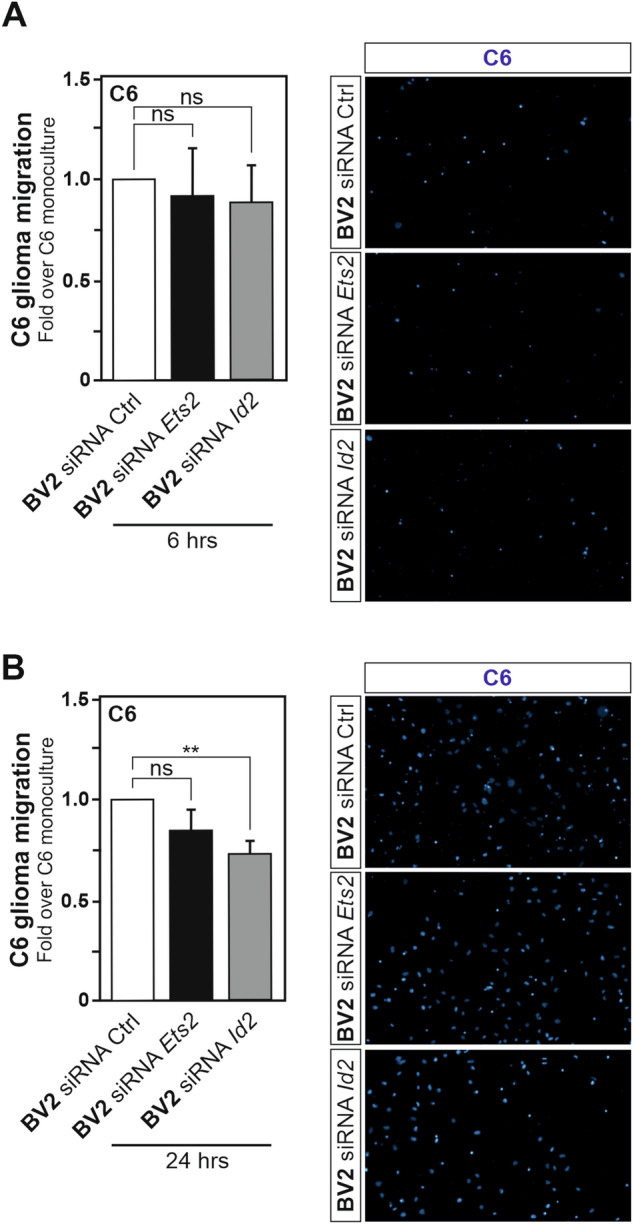
Perturbation of the ID2-ETS2 axis affects the acquisition of a microglial tumour-supportive phenotype. Quantification of the migration capacity at 6 (**A**) and 24 h (**B**) of C6 glioma cells in transwells alongside BV2 microglia in the lower compartment transfected with siRNA pools targeting *Id2* and *Ets2* gene expression. Results are presented relative to the migration of C6 glioma cells with BV2 microglia transfected with a non-targeting siRNA pool used as control, set as 1. Images presented in the right-hand side of each panel are used as representative illustrations of the migrated C6 cells for each condition. Data are presented as mean ± SEM. Statistics were performed with a Student’s t-test from 3 independent experiments. *P* value: **< 0.01, n.s. not significant for the indicated comparison.

Thereafter, the ability of BV2 microglia to enhance C6 glioma cells migration capability, as illustrated in Fig. 1A, was assayed upon *Id2* or *Ets2* gene silencing using siRNA pools in BV2 microglia (Fig. 5A, B). Reduction of microglial *Id2* gene expression levels was shown to significantly impair the migration capacity of C6 glioma cancer cells at 24 h using a transwell migration assay (Fig. 5B). Collectively, these data indicate that an ID2-ETS2 transcriptional axis contribute to the acquisition of a microglial tumour-supportive phenotype.

### Human glioblastoma tumour-associated microglia express *ID2* and *ETS2* genes

**Fig. 6:**
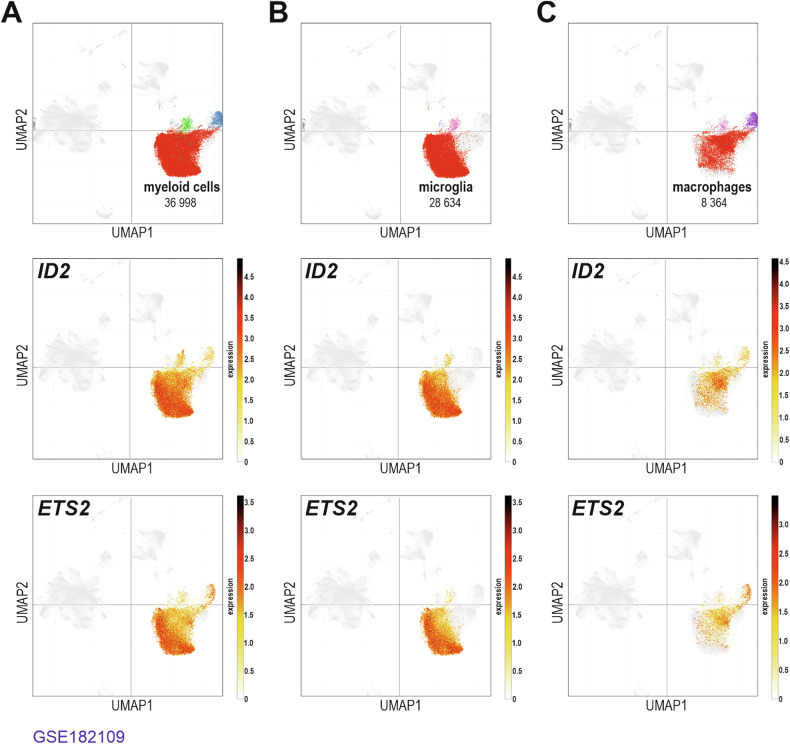
Human glioblastoma tumour-associated microglia/macrophages express *ID2* and *ETS2* genes. A publicly available single-cell RNA-sequencing dataset (Gene Expression Omnibus accession number: GSE182109) generated from generated from 18 glioma patients was reanalyzed. **A** T-distributed stochastic neighbour embedding (tSNE) plot representation of all myeloid single-sequenced cells from 11 newly diagnosed GB (36,998 cells), in combination with the analysis of *ID2* and *ETS2* gene expression. **B** Same analysis but only in microglia (28,634 cells) from newly diagnosed GB. **C** Same analysis but only in macrophages (8364 cells) from newly diagnosed GB.

In search of a pathophysiological relevance for the microglial transcriptional regulatory ID2-ETS2 axis we uncovered, we took advantage of an available human genome-wide gene expression dataset that investigated by single-cell RNA-sequencing in 18 glioma tumours, including 11 newly diagnosed GB, (characterized as IDH1-wildtype), the transcriptomes of the malignant neoplastic cells, and of the immune cells, including microglia and macrophages, within the tumour microenvironment [22]. For the newly diagnosed GB tumours, out of 94,450 single cells sequenced, 36,998 were labelled as belonging to the myeloid lineage; 28,634 cells were defined as brain resident microglia and 8,364 cells peripheral recruited bone-marrow-derived macrophages (Fig. 6A). Examples of gene expression commonly used to define microglia (*P2RY12* and *SALL1*) and peripheral recruited bone-marrow-derived macrophage (*MARCO* and *CD206*) are depicted in panel A and panel B, respectively, in Supplementary Fig. 2. In the newly diagnosed GB tumours, the neoplastic glioma cells show expression of genes that have been associated to the activation of microglia (*e.g*., *CCL2*, *CSF1*, *CX3CL1*, *CXCL12*, *CXCL16*, and *GDNF*), whereas the microglia show expression of genes associated to several tumour-supportive functions, *i.e*., ECM degradation and invasion (*e.g*., *CCL4*, *CCL5*, *CCL8*, *IL6*, *IL18*, *MMP2*, *MMP9*, *MMP14*, and *TGFB1*), immune suppression (*e.g*., *CCL2*, *IL10*, and *TGFB1*), proliferation and stemness (*e.g*., *CCL8*, *IL1B*, *IL10*, *PTN*, *STIP1*, and *TGFB1*), as well as angiogenesis (*e.g*., *CXCL2*, *IL6*, *MIF*, *TGFB1*, *VEGFA*, and *VEGFB*) (for reviews see [31, 32]) (Supplementary Fig. 3). Tumour-associated microglia/macrophages were found to express both *ID2* and *ETS2* genes, hence validating their expression in myeloid cells in the context of GB tumours (Fig. 6B, C). An additional human scRNA-seq dataset originating from 28 GB tumours further confirmed the expression of *ID2* and *ETS2* genes in tumour-associated microglia/macrophages in the context of GB tumours (Supplementary Fig. 4) [23].

### In glioma patients *ID2* and *ETS2* gene expression levels are associated with unfavourable respective favourable prognosis

**Fig. 7:**
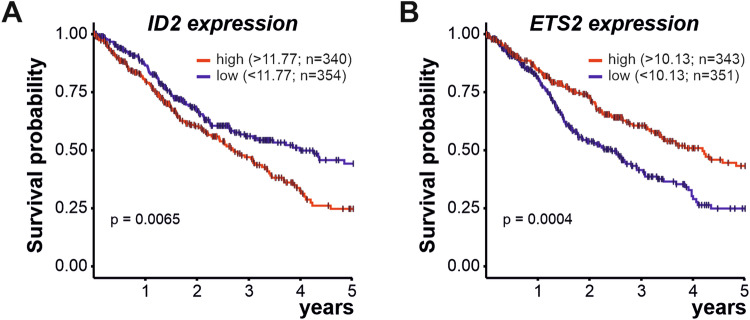
In glioma patients high *ID2* and *ETS2* gene expression are associated with unfavourable respective favourable prognosis. Gene expression levels for *ID2* (**A**) and *ETS2* (**B**) and clinical data extracted for a maximal 5 years period from the Cancer Genome Atlas (TCGA) for glioblastoma (TCGA-GBM) and lower-grade glioma (TCGA-LGG) cohorts were used to assess their potential prognostic values (raw data available in supplementary data file 2). High *versus* low expression (defined by median value) for *ID2* and *ETS2* genes were associated with unfavourable and favourable prognosis considering the 5 years overall survival, respectively (defined by days elapsed) for patients with glioma.

Since we observe that the ID2 (corepressor)-ETS2 (transcription factor) axis regulate the acquisition of the microglial tumour-supportive phenotype upon stimulation by glioma cancer cells, and that the expression of both *ID2* and *ETS2* genes were validated in tumour-associated microglia/macrophages isolated from glioblastoma tumours, we decided to investigate whether *ID2* gene expression levels, as well as the expression of the transcription factor regulated by ID2, *i.e*., *ETS2*, could be used as survival prognostic factors for patients suffering from glioma. For these two candidate genes, gene expression levels from bulk tumour RNA sequencing as well as corresponding clinical data were obtained from two combined human cohorts from The Cancer Genome Atlas (TCGA): TCGA-GBM, that including 617 GB cases and TCGA-LGG, that consist of 516 lower-grade glioma cases. From those, only 694 patients were included for survival analysis based on availability of both survival and expression data. Patient 5 years survival was analyzed by Kaplan–Meier method using R package survival. High and low gene expression groups were defined by median value and visualized employing survminer. Differences between the two groups were assessed for significance through log-rank test. Based on the log2(*x* + 1) transformed RSEM (RNA-Seq by expectation-maximization) normalized counts value of each gene, patients with glioma were classified into two expression groups, high and low gene expression groups defined based on the median value and the correlation between expression level and patient were classified into two expression groups, high and low gene expression groups defined based on the median value and the correlation between expression level and patient survival was examined. The prognosis of each group of patients was examined using Kaplan–Meier survival estimators, and the 5 years survival outcomes of the two groups were compared by log-rank tests. Supporting our findings, the analysis of these TGCA gene expression dataset revealed that *ID2* should be considered as an unfavourable prognostic gene (Fig. 7A) whereas *ETS2* as a favourable prognostic gene for patients with glioma (Fig. 7B).

## Discussion

Considering that the multiple biological functions exerted by microglia, including in the context of diseases, are associated with an equal number of gene expression profiles required to mediate these effects, the definition of transcriptional regulators for those transcriptomic programmes has recently gained interest [4, 7–13]. The identification of distinct regulators, controlling specific microglial transcriptional programmes, such as the tumour-supportive reactive state, could lead to the development of therapeutic strategies targeting specific microglial phenotypes, instead of the entire microglial cell population, which have been shown in mice to be unsuccessful at least to treat GB tumours [18]. Here, we report the identification in vitro of an ID2 (corepressor)-ETS2 (transcription factor) regulatory axis involved in the acquisition of a tumour-supportive phenotype by BV2 microglia once exposed to stimuli originating from C6 glioma cells. However, we cannot exclude that the components of this regulatory axis in the context of a brain neoplasm could also contribute to the regulation of the neoplastic cell biology.

ID2 belongs to the ID subfamily of HLH proteins known to antagonize the transcriptional activity of bHLH proteins. ID proteins, which lack the basic domain, form heterodimers with those transcription factors, thereby preventing their binding to E-box sequence on the promoter regions of target genes [26]. ETS proteins are transcription factors that share a conserved winged helix-turn-helix DNA binding domain (ETS domain), recognizing unique DNA sequences named ETS binding sites (EBS) [33]. ID proteins can also bind to the DNA-binding domain of ETS transcription factors and disrupt their recruitment to EBS [30].

The analysis of a publicly available single-cell transcriptomic dataset that included 11 adult GB tumours, and 94,450 single cells sequenced, indicates that while *ID2* gene expression was found to be significantly expressed in both the myeloid and the neoplastic cell compartments, *ETS2* gene expression levels appears to be higher in the myeloid cells [22]. Likewise, the preferential expression of *ETS2* in tumour-associated microglia/macrophages, in a glioblastoma tumour context, as compared to *ID2* being expressed in myeloid and neoplastic cells, was also observed in two other RNA-sequencing datasets consisting of 3589 single cells originating from four adult GB tumours, and 7930 single cells originating from 28 adult and paediatric GB tumours [12, 23].

Several pro-tumoral functions have been reported for the ID2 transcription regulator in GB cells. Elevated ID2 expression can promote their survival to glucose deprivation, possibly *via* the regulation of the expression of genes encoding for components of the mitochondrial electron transport chain complexes that in turn result in reduced mitochondrial superoxide and reactive oxygen species productions, as well as reduced mitochondrial damages [34]. In addition, ID2 expression in U-87MG GB cells (RRID:CVCL_0022), promotes cell migration through the repression of bHLH transcription factor 3 (TCF3)-induced semaphorin 3F (SEMA3F) expression, SEMA3F being a potent inhibitor of metastasis [35, 36]. In contrast, the downregulation of *ID2* gene expression by RNA interference in a panel of human GB cell lines, including U-87MG, increased their sensitivity to anticancer drugs such as temozolomide, as illustrated by an enhanced occurrence of apoptosis in those cells [37]. The ID2 small molecule inhibitor, AK-778-XXMU, is able to decrease the migration capability of U-87MG GB cells, and to increase their apoptotic cell death in vitro. In vivo, when U-87MG GB cells are transplanted subcutaneously in NCG mice, AK-778-XXMU treatment significantly reduce tumour growth [38]. However, it should be noted that the use of AK-778-XXMU, as well as Helichrysetin, another candidate ID2 inhibitor isolated from the Rwandese medicinal plant *Helichrysum odoratissimum* [39], to treat brain neoplasm such as GB remain questionable. Indeed, in silico analyses reveal that both drugs have poor physicochemical properties (1) polar surface area; (2) number of hydrogen donors; (3) log P saying about lipohilicity; and (4) molecular weight to passively penetrate the blood brain barrier, and consequently only limited plausible brain bioavailability [40–42]. Hence, collectively these studies indicate that ID2 affects the malignant progression of GB through different mechanisms. However, they do not address the possible contribution of ID2 to the regulation of biological functions associated to tumour-associated myeloid cells in the context of GB.

As its former name indicates, *i.e*., inhibitor of differentiation 2, ID2 has been reported to be involved in the differentiation of multiple cell types, including from the myeloid cell lineage. In human, the *ID2* gene is found to be constitutively expressed in more mature myeloid cells and its expression level markedly increases during the terminal myeloid differentiation of bone-marrow-derived cells [43]. In mice, during foetal liver development, the retinoblastoma (RB) transcriptional corepressor 1 (RB1) promotes the differentiation of macrophages by opposing the inhibitory functions of ID2 on the ETS transcription factor family member, Spi-1 proto-oncogene (SPI1, also known as PU.1), a master regulator of myeloid cell differentiation [44]. However, also in mice, while the differentiation of microglia from erythromyeloid precursor originating from the yolk sac during embryonic development was found to depend on SPI1/PU.1, ID2 was not required [6]. Yet, our investigation reveals that increased *ID2* gene expression is linked to the acquisition of a glioma cells-induced tumour-supportive reactive state by microglia, and that microglia, as well as macrophage, in the context of a GB tumour are also associated with a high *ID2* gene expression. Repression of *Id2* gene expression in microglia, in vitro, was associated with a reduced capacity for these cells to promote glioma cell migration. Other microglial functions regulated by ID2 remains to be elucidated. For example, ID2 is reported to promote the proangiogenic functions of bone-marrow-derived myeloid cells that infiltrate HGG, through the inhibition of TCF3-induced *KDR* (kinase insert domain receptor, also known as *VEGFR2*) gene expression [45]. Collectively, our data and the existing literature suggest that in the context of brain neoplasm like GB, the transcriptional regulator ID2 can contribute positively to tumour progression, exerting effects on the malignant cells themselves, as well as myeloid cells, such as microglia within their tumour microenvironment.

As mentioned above, single-cell transcriptomic datasets indicate that *ETS2* gene expression is predominantly restricted to the myeloid cell compartment within the GB tumour mass. Therefore, it would be of interest to understand whether ETS2 acting as a transcription factor regulates a transcriptional programme in tumour-associated myeloid cells that is potentially associated with an immune reactive phenotype holding anti-tumoral properties, which would be repressed by the induction of ID2 expression in those cells. In fact, ETS2 eases cytokines production, *e.g*., interleukin-6 (IL6), tumour necrosis factor (TNF, also known as TNF-alpha), and interferon beta 1 (IFNß1), initiated by the activation of Toll-like receptors (TLR) in macrophages, through the suppression of the MAPK/NF-κB signalling, recruitment of histone deacetylase 1 (HDAC1), key regulators of the pro-inflammatory response in both macrophages and microglia [46, 47]. In mammary tissue, ETS2 is also reported to regulate a transcriptomic programme that includes genes encoding for inhibitors of angiogenesis [48]. As a result, in mice, the deletion of *Ets2* in macrophages leads to exacerbated cytokines production upon a TLR challenge, but reduced lung metastasis in breast tumour models [46, 48].

Hence, the transcription factor ETS2 revealed itself as a key regulator of macrophage functions associated with tumour suppression. Likewise, our result suggests that ETS2 could exert similar functions in microglia, which could be repressed by the induction of ID2 expression in those cells when they are recruited and converted into tumour-trophic cells by the glioma cancer cells. Supporting the idea of a pro-tumoral effect associated with ID2 expression, and inversely anti-tumoral property linked to ETS2 expression in GB tumours, high *ID2* and *ETS2* gene expression levels were found to be associated respectively with poor and good prognosis in cohort of patients with gliomas. Future therapeutic interventions aimed at manipulating the ID2-ETS2 axis could potentially alter the dynamics of the tumour microenvironment, providing new avenues for enhancing treatment outcomes in patients with glioblastoma.

### Supplementary information

Supplementary figures and Data files legends

Supplementary Figure 1

Supplementary Figure 2

Supplementary Figure 3

Supplementary Figure 4

original data

Supplementary data File 2

Supplementary Tables 1 to 3

## Data Availability

All data presented in the study are available from the corresponding author upon reasonable request. Part of the data that support the findings of this study are openly available in TCGA repository and at the Single Cell Portal. https://singlecell.broadinstitute.org/single_cell/study/SCP1985/single-cell-analysis-of-human-glioma-and-immune-cells-identifies-s100a4-as-an-immunotherapy-target-gse182109. https://singlecell.broadinstitute.org/single_cell/study/SCP393/single-cell-rna-seq-of-adult-and-pediatric-glioblastoma#study-visualize. https://portal.gdc.cancer.gov/projects/TCGA-GBM. https://portal.gdc.cancer.gov/projects/TCGA-LGG.
