# Supplementary material for: ID2-ETS2 axis regulates the transcriptional acquisition of pro-tumoral microglia phenotype in glioma
Source: Cell Death Dis. 2024 Jul 18;15(7):512. doi: 10.1038/s41419-024-06903-3 (PMC11255298; doi:10.1038/s41419-024-06903-3)
Supplement: Supplementary file 1 — Supplementary figures and Data files legends [file 41419_2024_6903_MOESM1_ESM.docx]

**Supplementary figures and Data files legends**

**Supplementary fig. 1 | Validation of *Id2* and *Ets2* gene silencing by small interfering RNA pools.**

*Id2* (**A**), *Id1* (**B**) and *Ets2* (**C**) mRNA expression measured by RT-qPCR in BV2 microglia transfected with siRNAs pool targeting *Id2* (a and b) or *Ets2* (c) gene expression, for 24 hours as compared to BV2 microglia transfected with control non-targeting siRNAs pool, set as 1. **D**) Immunoblot analysis of ETS2 protein expression in BV2 cells transfected with *Ets2* siRNAs pool or with control non-targeting siRNAs pool. The expression of ACTB as housekeeping gene was used as loading control. **E**) Quantification of ETS2 protein expression level, BV2 microglia transfected with control non-targeting siRNAs pool, set as 1. Data are presented as mean ± SEM. Statistics were performed with a Student’s t-test from 3 independent experiments. P value * < 0.05, ** < 0.01, ***<0.001 n.s., not significant for the indicated comparison.

**Supplementary fig. 2 | Expression of genes associated to microglia versus bone marrow derived macrophages in the human single-cell RNA-sequencing dataset** (GSE182109)**.**

**A**) T-distributed stochastic neighbour embedding (tSNE) plot representation of microglia (28,634 cells) from newly diagnosed GB in combination with the analysis of *MARCO, CD209* (BMDM markers), as well as *P2RY12 and SALL1* (microglia markers) gene expression. **B**) Same analysis but only in macrophages (8,364 cells) from newly diagnosed GB.

**Supplementary fig. 3 | Expression of genes associated to microglial tumour-supportive functions in the human single-cell RNA-sequencing dataset** (GSE182109)**.**

**A**) T-distributed stochastic neighbour embedding (tSNE) plot representation of neoplastic cells (40,019 cells) from newly diagnosed GB in combination with the analysis of expression of genes that have been associated to the activation of microglia (*CCL2*, *CSF1*, *CX3CL1*, *CXCL12*, *CXCL16*, and *GDNF*). **B-E**) tSNE plot representation of microglia (28,634 cells) from newly diagnosed GB in combination with the analysis of expression of genes associated to several tumour supportive functions, *i.e*., (B) ECM degradation and invasion (*e.g*., *CCL4*, *CCL5*, *CCL8*, *IL6*, *IL18*, *MMP2*, *MMP9*, *MMP14*, and *TGFB1*), (C) immune suppression (*e.g*., *CCL2*, *IL10*, and *TGFB1*), (D) proliferation and stemness (*e.g*., *CCL8*, *IL1B*, *IL10*, *PTN*, *STIP1*, and *TGFB1*), and (D) angiogenesis (*e.g*., *CXCL2*, *IL6*, *MIF*, *TGFB1*, *VEGFA*, and *VEGFB*).

**Supplementary fig. 4 | Expression of *ID2* and *ETS2* genes in the human single-cell RNA-sequencing dataset** (GSE131928)**.**

A publicly available single-cell RNA-sequencing dataset generated from 28 human glioblastoma tumour biopsies was reanalysed. **A**) T-distributed stochastic neighbour embedding (tSNE) plot representation of all single sequenced cells. Cells are coloured based on expression of sets of marker genes for tumour cells (blue), myeloid cells (red), oligodendrocytes (green) and T cells (purple). In the single-cell RNA sequencing dataset. **B**) Expression of *ID2* and **C**) *ETS2* genes, validating expression in myeloid cells in the context of glioblastoma tumours.

**Supplementary Data file 1 | Immunoblot.**

Full and uncropped immunoblots for supplementary figure 1E. Lanes 5 and 6 are depicted in the figure.

**Supplementary Data file 2 | TCGA raw data.**

Gene expression levels for *ID2* and *ETS2* and clinical data extracted for a maximal 5 years period from the Cancer Genome Atlas (TCGA) for glioblastoma (TCGA-GBM) and lower-grade glioma (TCGA-LGG) cohorts.
