# Supplementary material for: ID2-ETS2 axis regulates the transcriptional acquisition of pro-tumoral microglia phenotype in glioma
Source: Cell Death Dis. 2024 Jul 18;15(7):512. doi: 10.1038/s41419-024-06903-3 (PMC11255298; doi:10.1038/s41419-024-06903-3)
Supplement: Supplementary file 4 — Supplementary Figure 3 [file 41419_2024_6903_MOESM4_ESM.pdf]

Supplementary Figure 3.

A **Glioma factors leading to microglia activation**

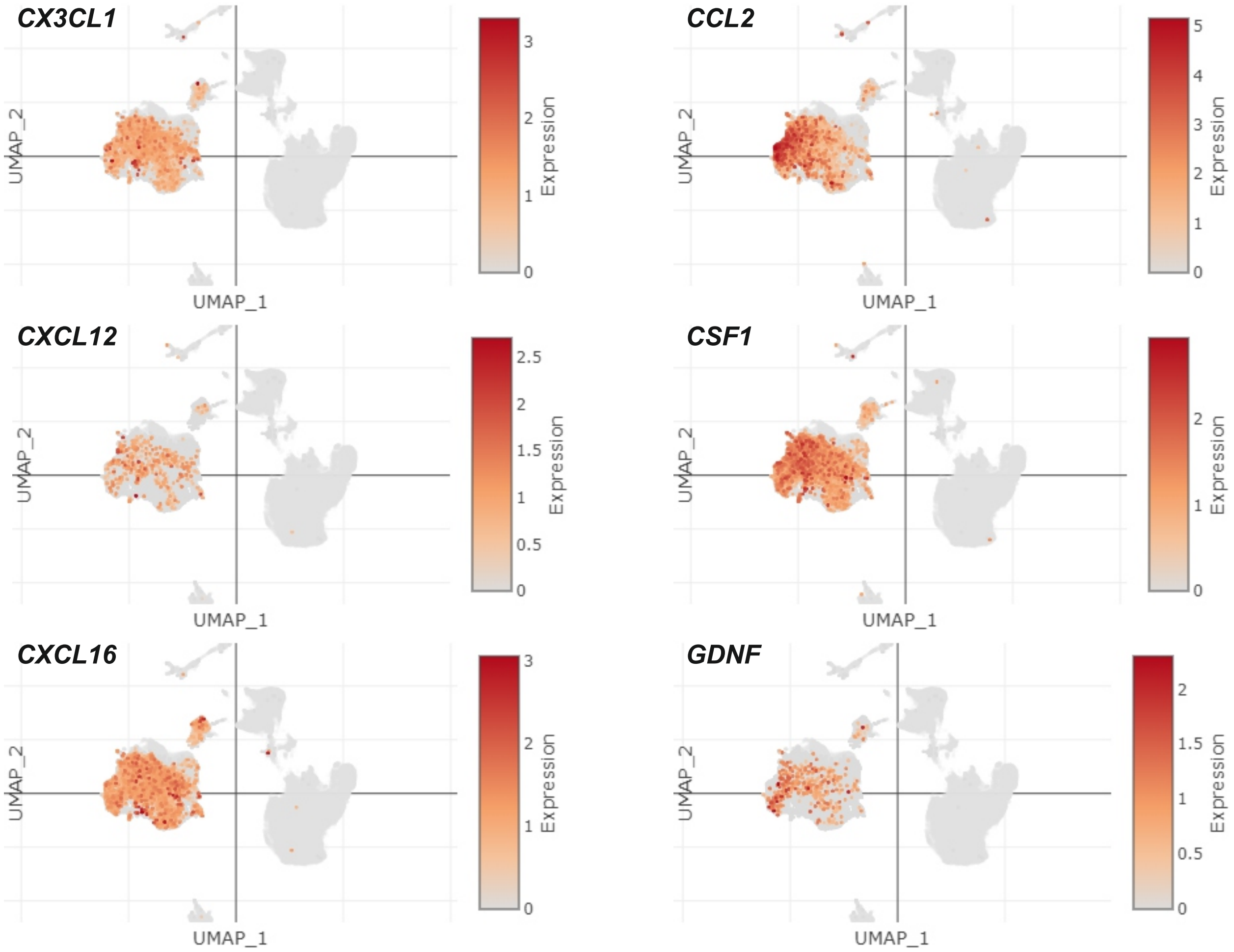

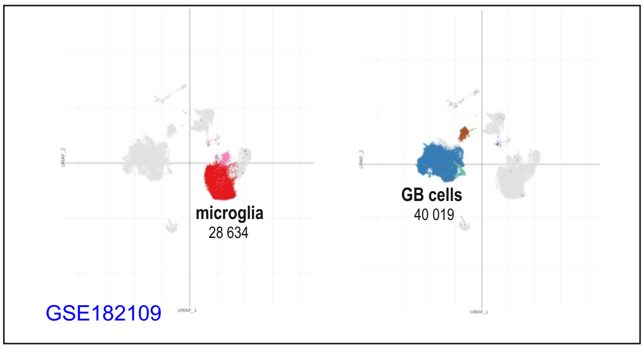

B ## ECM degradation and invasion

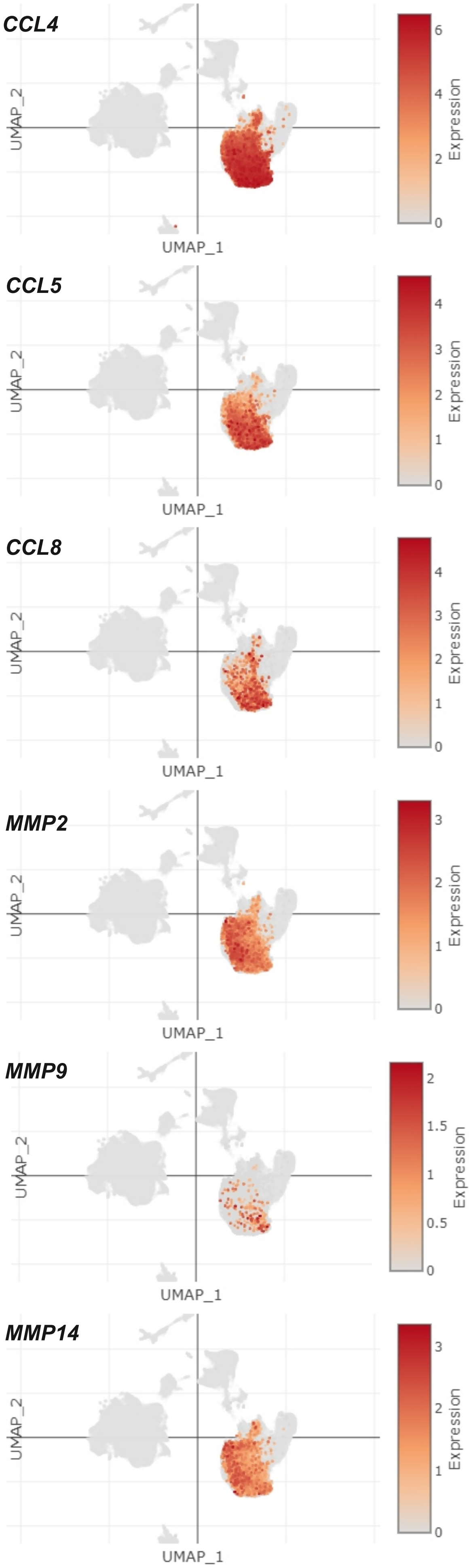

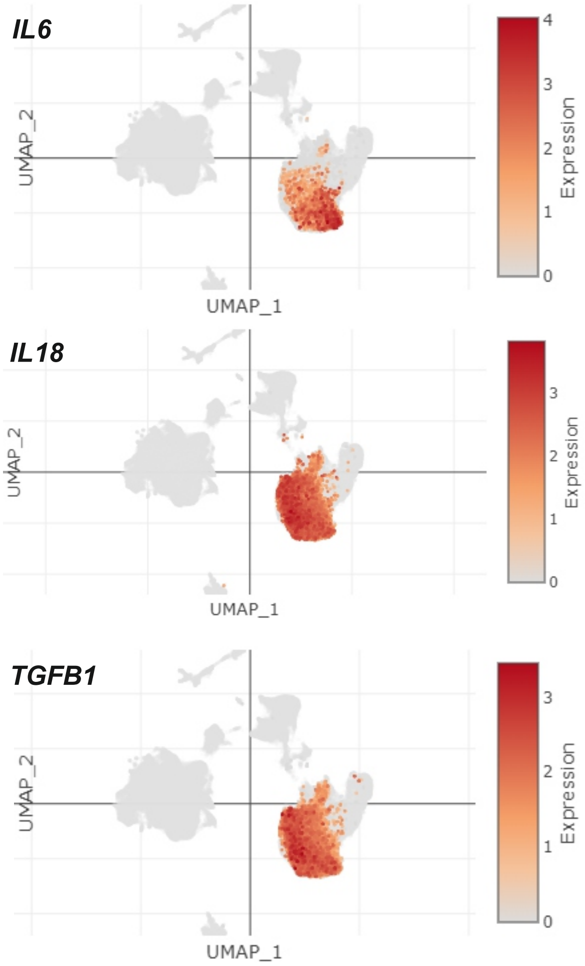

C ## Immune supression

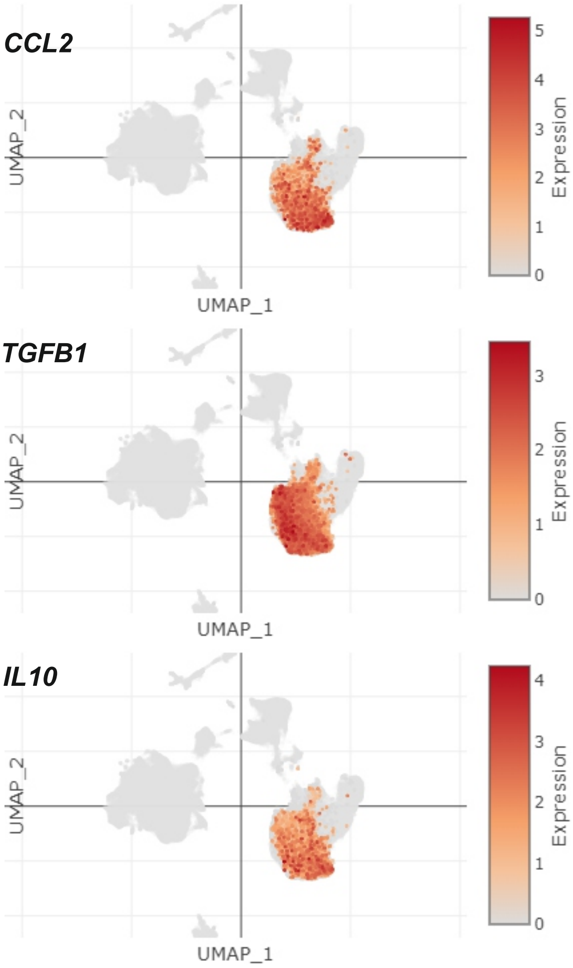

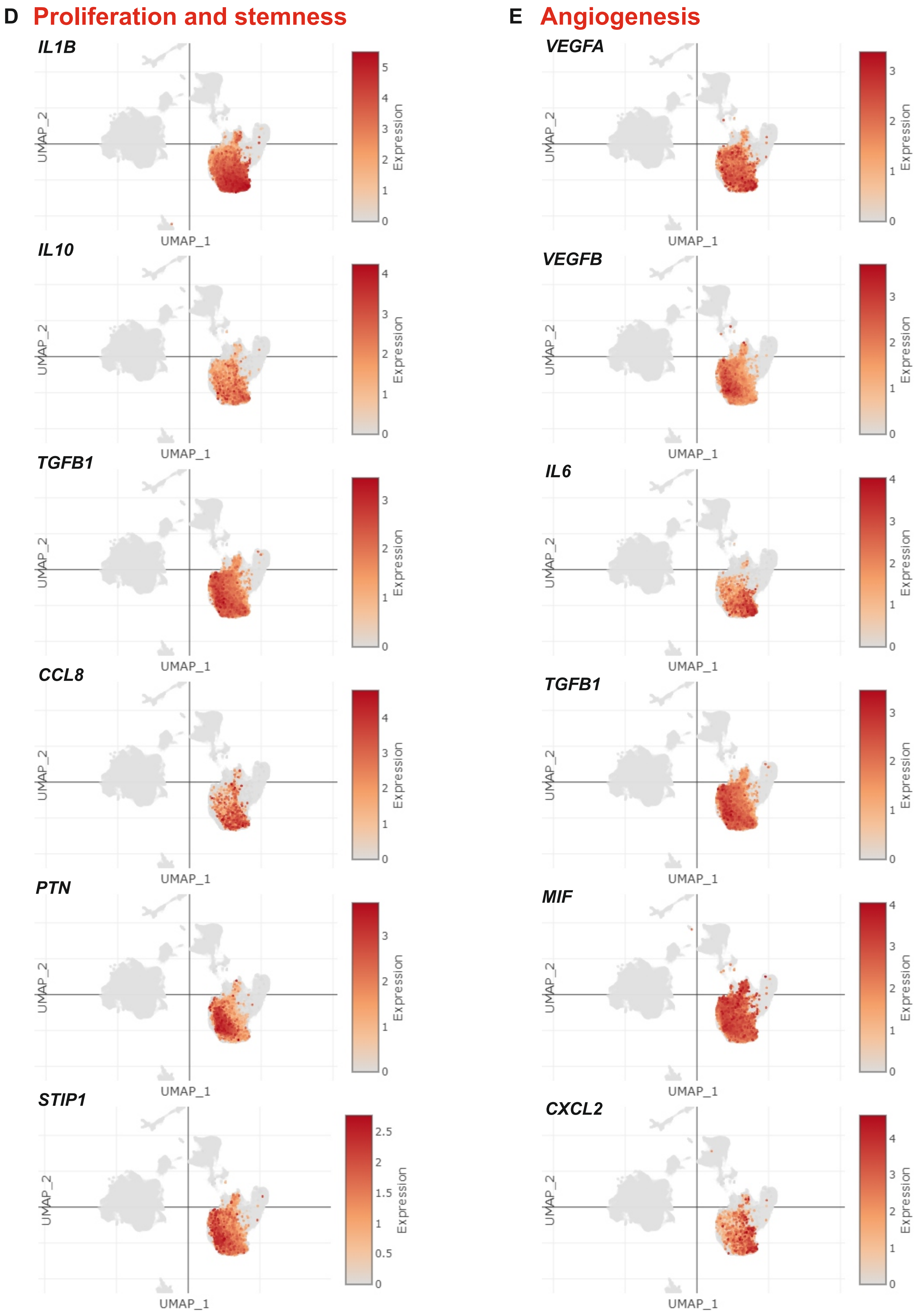

D
Proliferation and stemness
IL1B
IL10
TGFB1
CCL8
PTN
STIP1
E
Angiogenesis
VEGFA
VEGFB
IL6
TGFB1
MIF
CXCL2
UMAP_1
UMAP_2
Expression
