# Supplementary figures and images for: ID2-ETS2 axis regulates the transcriptional acquisition of pro-tumoral microglia phenotype in glioma
Source: Cell Death Dis. 2024 Jul 18;15(7):512. doi: 10.1038/s41419-024-06903-3 (PMC11255298; doi:10.1038/s41419-024-06903-3)

**Supplementary Data fie 1.**

original WB

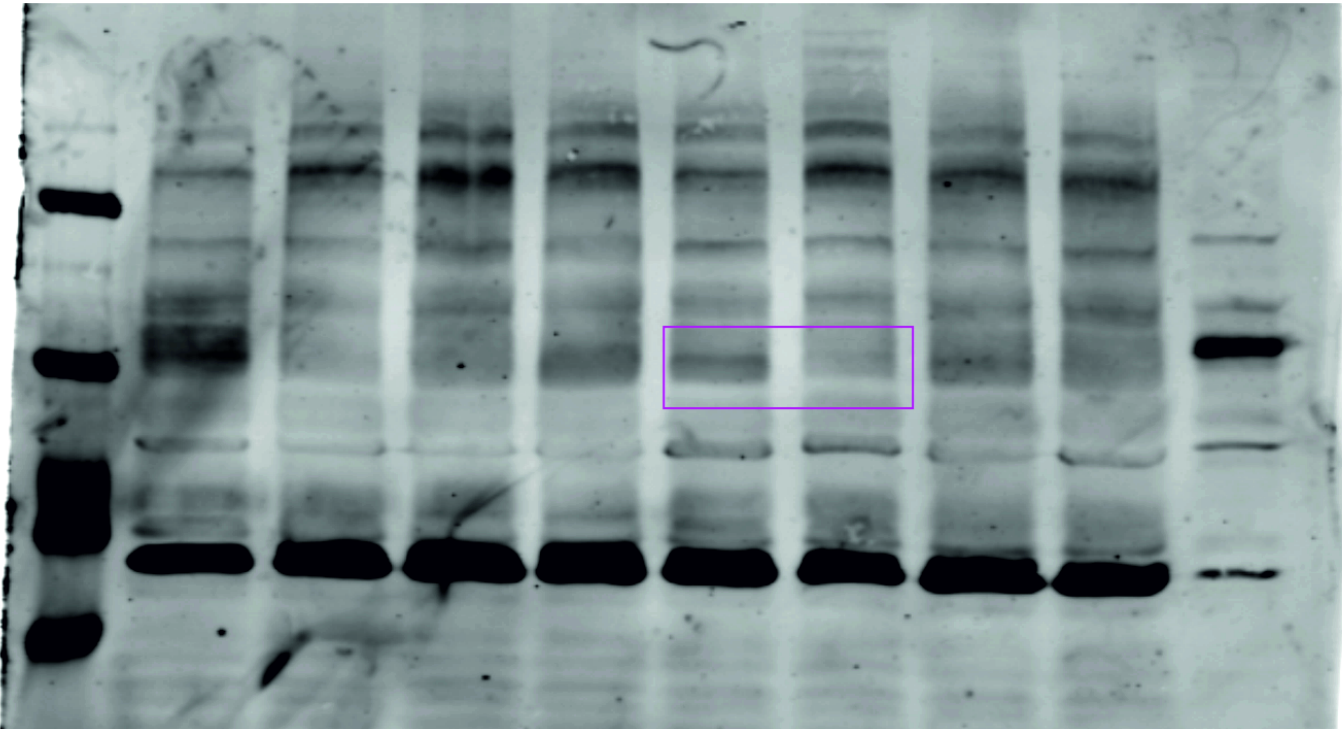

**ETS2**

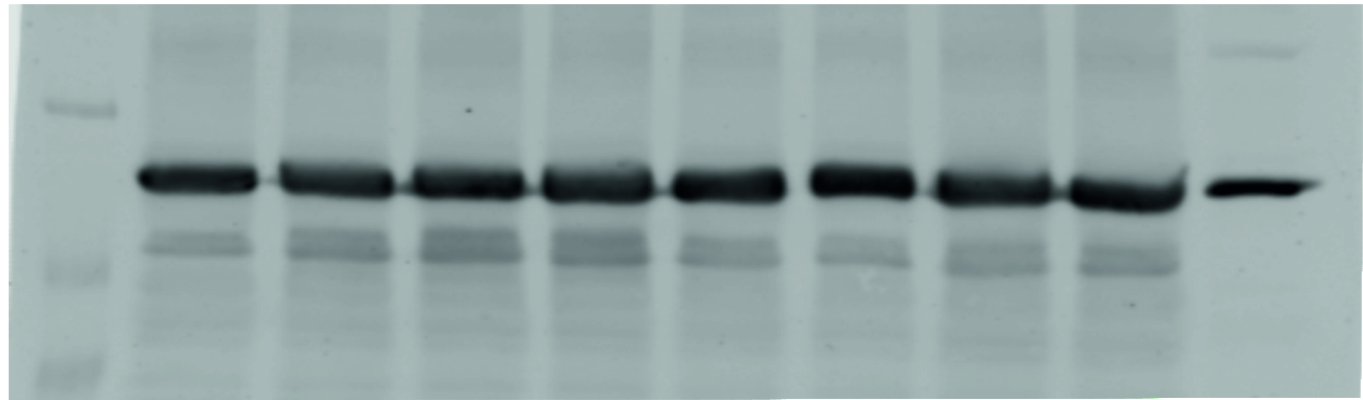

**ACTB**

Supplement: Supplementary file 6 — original data [file 41419_2024_6903_MOESM6_ESM.pdf]
