# Supplementary material for: ID2-ETS2 axis regulates the transcriptional acquisition of pro-tumoral microglia phenotype in glioma
Source: Cell Death Dis. 2024 Jul 18;15(7):512. doi: 10.1038/s41419-024-06903-3 (PMC11255298; doi:10.1038/s41419-024-06903-3)
Supplement: Supplementary file 8 — Supplementary Tables 1 to 3 [file 41419_2024_6903_MOESM8_ESM.docx]

**Supplementary Table 1 | Primer sequences**

All sequences are given 5´to 3´

| **cDNA** (organism) | | **Forward primer** | **Reverse primer** |
| --- | --- | --- | --- |
|  |  | |  |
| ***Actb*** (mouse) | GATGTATGAAGGCTTTGGTC | | TGTGCACTTTTATTGGTCTC |
| ***Dusp6*** (mouse) | CTCGGATCACTGGAGCCAAAAC | | TCTGCATGAGGTACGCCACTGT |
| ***Ets2*** (mouse) | GTCAATTACTGTTCCATCAGC | | CCTTGGGTTTTCCAGAATTG |
| ***Fli1*** (mouse) | CCATACAGACCAGTCCTCACGA | | CATGGTCTGTGATCCTCCAAGG |
| ***Hmox1*** (mouse) | CACTCTGGAGATGACACCTGAG | | GTGTTCCTCTGTCAGCATCACC |
| ***Id1*** (mouse) | ATCTCTGGGAAAGACACTAC | | ATAAAACAGAAACACGCGG |
| ***Id2*** (mouse) | TCTGTGGCTAAATAAATGGC | | CGATCATCCTTAGTTTTCCTTC |
| ***Jun*** (mouse) | CAGTCCAGCAATGGGCACATCA | | GGAAGCGTGTTCTGGCTATGCA |
| ***Stab1*** (mouse) | TCCGCTGTACTCAAGGCTTCCA | | CTTCTTGGCACAGGTGTAGGAAC |
| ***Il1b*** (mouse) | ACCAAGCAACGACAAAATAC | | CACTTTGCTCTTGACTTCTATC |
| ***Mmp9*** (mouse) | GTTTTCTTCTTCTCTGGACG | | CTAGACCCAACTTATCCAGAC |
| ***Vegfβ*** (mouse) | GCCACCAGAAGAAAGTGGTGC | | CTGGGCACTAGTTGTTTGACC |
| ***Tgfβ1*** (mouse) | TGCGCTTGCAGAGATTAAAA | | CGTCAAAAGACAGCCACTCA |
|  |  | |  |

**Supplementary Table 2 | Antibodies used in this study.**

When available, reference number to 1DegreeBio antibody validation profile is indicated.

| **Primary Antibodies** | **Company** (Cat#, RRID) | **Dilution used** |
| --- | --- | --- |
|  |  |  |
| **ETS2** (rabbit pAb) | GeneTex  (Cat# GTX104527,  RRID: AB_11179034) | 1/200 (PLA)  1/500 (WB) |
| **ID1** (B8, mouse mAb) | Santa Cruz Biotechnology (Cat# sc-133104,  RRID: AB_2122863) | 1/400 (PLA) |
| **ID2** (E7, mouse mAb) | Santa Cruz Biotechnology (Cat# sc-398104,  RRID: AB_2943636) | 1/50 (PLA) |
| **ACTB** (AC40; mouse mAb) | Sigma-Aldrich  (Cat# A3853,  RRID: AB_262137) | 1/2000 (WB) |
|  |  |  |
| **Secondary Antibodies** | **Company** (Cat#, RRID) | **Dilution used** |
|  |  |  |
| **IRDye® 800CW** (Goat anti-Mouse) | LI-COR Biosciences  (Cat# 926-32210,  RRID: AB_621842) | 1/5000 (WB) |
| **IRDye® 680RD** (Goat anti-Rabbit) | LI-COR Biosciences  (Cat# 926-68071,  RRID: AB_10956166) | 1/5000 (WB) |
|  |  |  |
|  |  |  |

**Supplementary Table 3 |ON-TARGETplus SMART pool small interfering RNAs used in this study.**

| **ON-TARGET plus SMARTpools siRNAs** | **Company** |
| --- | --- |
|  |  |
| ***Id2*** (mouse, NM_010496) | Dharmacon (L-060495-00-0005) |
| CUUCUGAGCUUAUGUCGAA |  |
| GCAAAGUACUCUGUGGCUA |  |
| GGUGAGGUCCGUUAGGAAA |  |
| CCAAAUAAAUACCAGUUCA |  |
|  |  |
| ***Ets2*** (mouse, NM_011809) | Dharmacon (L-040983-01-0005) |
| GAACUGGCUUCGCCGUUUA |  |
| UCCACAAGACUUCGGGCAA |  |
| ACUGAGAGAAACCGUGAUA |  |
| CGUUAUACCAUGAGACCAC |  |
|  |  |
| **Non-targeting siRNA pool** | Dharmacon (D-001810) |
| UGGUUUACAUGUCGACUAA |  |
| UGGUUUACAUGUUGUGUGA |  |
| UGGUUUACAUGUUUUCUGA |  |
| UGGUUUACAUGUUUUCCUA |  |
